# Multiple antibiotic susceptibility of polyphosphate kinase mutants (*ppk1* and *ppk2*) from *Pseudomonas aeruginosa* PAO1 as revealed by global phenotypic analysis

**DOI:** 10.1186/s40659-015-0012-0

**Published:** 2015-04-25

**Authors:** Javiera Ortiz-Severín, Macarena Varas, Catalina Bravo-Toncio, Nicolás Guiliani, Francisco P Chávez

**Affiliations:** Systems Microbiology Laboratory, Department of Biology, Faculty of Science, University of Chile, Las Palmeras 3425, Ñuñoa, Santiago, Chile; Bacterial Communication Laboratory, Department of Biology, Faculty of Science, University of Chile, Las Palmeras 3425, Ñuñoa, Santiago, Chile

**Keywords:** Antibiotic susceptibility, Polyphosphate kinase, Pseudomonas, Phenome, Phenotypic microarrays, Multidrug resistance

## Abstract

**Background:**

*Pseudomonas aeruginosa* is known to be a multidrug resistant opportunistic pathogen. Particularly, *P. aeruginosa* PAO1 polyphosphate kinase mutant (*ppk1*) is deficient in motility, quorum sensing, biofilm formation and virulence.

**Findings:**

By using Phenotypic Microarrays (PM) we analyzed near 2000 phenotypes of *P. aeruginosa* PAO1 polyP kinase mutants (*ppk1* and *ppk2*). We found that both *ppk* mutants shared most of the phenotypic changes and interestingly many of them related to susceptibility toward numerous and different type of antibiotics such as Ciprofloxacin, Chloramphenicol and Rifampicin.

**Conclusions:**

Combining the fact that *ppk1* mutants have reduced virulence and are more susceptible to antibiotics, polyP synthesis and particularly PPK1, is a good target for the design of molecules with anti-virulence and anti-persistence properties.

**Electronic supplementary material:**

The online version of this article (doi:10.1186/s40659-015-0012-0) contains supplementary material, which is available to authorized users.

## Findings

*Pseudomonas aeruginosa* is a major opportunistic pathogen frequently involved in hospital-acquired infections and can produce severe pneumonia, burn wound infections, and sepsis. Particularly, multidrug resistant (MDR) variants are emerging rapidly in the clinic for this pathogen. In addition, *P. aeruginosa* resistance rates have increased to available antimicrobial agents, limiting the choice of available anti-infective chemicals [1]. Looking for alternatives with economic and human health impact, new antimicrobial agents with novel biological targets or strategies are desperately needed to combat highly resistant *P. aeruginosa* infections [2,3]. Current original strategies combine the reduction of bacterial virulence with a simultaneous increase of animal host defence, instead of eradicating the pathogen [4,5].

Inorganic polyphosphate (polyP) are essential for bacterial resilience during stress and stringencies, cellular motility, biofilm formation and virulence [6]. Many bacterial pathogens knockout of polyP synthesis gene (*ppk1*) result in cellular defects, particularly in the context of virulence toward the host they invade [6,7]. Specifically, a *Pseudomonas aeruginosa* PAO1 mutant of polyP synthesis (*Pappk1*) was impaired in motility, biofilm development, quorum sensing and virulence in ocular and burned-mouse models [8,9]. Moreover, this mutant exhibited reduced viability after exposure to a β-lactam antibiotic [10]. Similar results were reported in *ppk1* mutants from *S. typhimurium* and *S. dublin* that used Polymyxin B15 antibiotic [11]. In addition to homologues of PPK1, another widely conserved polyP enzyme is PPK2, which, in contrast to the ATP-dependent polyP synthetic activity of PPK1, preferentially catalyses the reverse reaction, polyP-driven synthesis of GTP from GDP [12]. The fact that polyP is involved in bacterial virulence and resilience processes makes it an attractive target for antimicrobial agents [2].

To provide a more complete analysis of *P. aeruginosa* polyP synthesis mutants (*ppk1* and *ppk2*) phenotypes and to obtain greater insight into the physiological changes and particularly chemical susceptibility we used Phenotype Microarray (PM) technology [13]. Biolog Phenotype MicroArrays studies in *Pseudomonas aeruginosa* PAO1 have been used to facilitate further characterization of known mutation strains and for testing bioinformatic predictions for mutations in hypothetical or unknown genes [14]. We used *P. aeruginosa* polyphosphate synthesis knockout mutants *ppk1* (PA5242) and *ppk2* (PA0141) from a *P. aeruginosa* Mini- Tn5-Tcr gene knockout mutants collection [15]. We confirmed by PCR both mutations and bacterial cell suspensions from all strains were inoculated into each of the 20 PM plates for full metabolic profiling according to standard protocols recommended by Biolog Inc. for *Pseudomonas* strains [14,16]. The PM plates were located in an aerobic OmniLog incubator reader set at 30°C which collected data every 15 min over a 72-h period. PM tests were conducted in duplicate, and the plates were also examined visually at the end of each incubation period for independent confirmation. The OmniLog® V. 1.5 comparison module and the average height parameter were used for data analysis with standard thresholds for detection. A consensus graphical profiles for all metabolic and sensitivity tests for each mutant were generated using two independent runs (Additional file 1: Figure S1).

For visualization and clustering data analysis, Multiexperiment Viewer (MeV version 4.6) software was used. MeV is part of the TM4 Microarray Software Suite, an open source system for statistical and clustering analysis of omics data [17].

**Figure 1:**
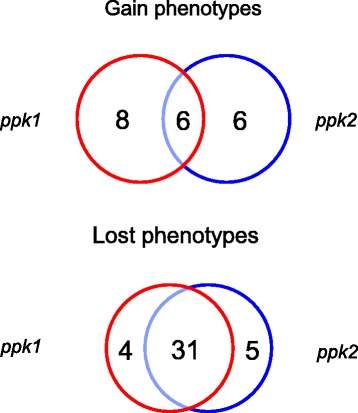
Venn Diagrams derived from the Phenotype MicroArrays results of *P. aeruginosa* PAO1 polyP synthesis mutants. The numbers indicate the total phenotypes gained or lost for the wild type strain compared with the mutant strains *ppk1* (left) and *ppk2* (right).

Comparing the metabolic and sensitivity capabilities between mutants and their isogenic parent strains we show that both mutant strains behave similar in nutrient utilization (PM1-10) and sensitivity to chemicals (PM11-20) assays (Figure 1, Table 1). This indicates that these two strains shared most of the phenotypic patterns and differed only in a few features (Figure 1, Additional file 2: Tables S1-S4). Particularly interesting is the altered isoleucine metabolism found exclusively in the *ppk1* mutant (Additional file 2: Table S2). In *Pseudomonas* persistence of *ppk1* mutant in the stationary phase was significantly affected [18,19]. In contrast, the main metabolic pathways were not significantly influenced by the loss of *ppk1* as revealed from respiration patterns of the *ppk* mutants in phenotypic microarrays. Our results are in agreement with those reported in *Pseudomonas putida* KT2440 where accumulation of inorganic polyphosphate enables stress endurance and catalytic vigour but the major metabolic routes were not significantly influenced by the loss of *ppk1* [18].

**Table 1:** **Summary of** ***P. aeruginosa*** **PAO1 polyP synthesis mutants (** ***ppk1*** **and** ***ppk2*** **) common gain and lost phenotypes**

**Test**	***ppk1*** **difference**	***ppk2*** **difference**	**Mode of action**
	**Gain phenotype**	**Gain phenotype**	
L-Arginine	50	75	C-Source, carboxylic acid
His-Met	61	56	N-Source, peptide
Carbenicillin	158	71	Wall, lactam
Cefsulodin	80	80	Wall, cephalosporin
Ruthenium red	78	110	Respiration, mitochondrial Ca^2+^ porter
Erythromycin	67	80	Protein synthesis, 50S ribosomal subunit, macrolide
	**Lost phenotype**	**Lost phenotype**	
D-Gluconic acid	−112	−106	C-Source, carboxylic acid
D-Mannitol	−68	−57	C-Source, carbohydrate
L-Valine	−60	−58	N-Source, amino acid
L-Methionine Sulfone	−69	−95	S-Source, organic
Orphenadrine	−78	−146	Anti-cholinergic
Patulin	−534	−495	Microtubulin polymerization inhibitor, antifungal
2,2′-Dipyridyl	−401	−397	Chelator, lipophilic
Sodium Arsenite	−332	−242	Toxic anion
Sodium Arsenate	−305	−321	Toxic anion, P04 analog
Dichlofluanid	−271	−119	Fungicide, phenylsulphamide
Thiamphenicol	−269	−193	Protein synthesis, amphenicol
Chloramphenicol	−209	−173	Protein synthesis, amphenicol
Chlorodinitrobenzene	−182	−187	Oxidizes sulfhydryls, depletes glutathione
8-Hydroxyquinoline	−181	−230	Chelator, lipophilic
Nafcillin	−163	−172	Wall, lactam
Antimony (III) chloride	−158	−153	Toxic cation
1,10-Phenanthroline monohydrate	−152	−171	Chelator, lipophilic
Captan	−152	−113	Fungicide, carbamate
Sulfathiazole	−148	−172	Folate antagonist, PABA analog
Gallic acid	−141	−115	Respiration, ionophore, H^+^
Poly-L-lysine	−140	−63	Membrane, detergent, cationic
Protamine sulfate	−129	−109	Membrane, nonspecific binding
Sulfadiazine	−127	−107	Folate antagonist, PABA analog
Oxacillin	−124	−130	Wall, lactam
Myricetin	−113	−122	DNA & RNA synthesis, polymerase inhibitor
Nickel chloride	−113	−80	Toxic cation
Plumbagin	−106	−118	Oxidizing agent
Oxophenylarsine	−99	−161	Tyrosine phosphatase inhibitor
trans-Cinnamic acid	−98	−90	Respiration, ionophore, H^+^
EDTA	−67	−88	Chelator, hydrophilic
Glycine hydroxamate	−66	−136	tRNA synthetase

The respiration patterns of *P. aeruginosa* PAO1 *ppk1* and *ppk2* associated with the metabolism of carbon (PM1-PM2), nitrogen (PM3), phosphorous and sulfur (PM4) sources were relatively moderate as compared to previous unpublished PM results from our laboratory with *Δppk1* mutant from *Escherichia coli* (Additional file 1: Figures S2 and S3). We speculate that the apparent metabolic robustness in *Pseudomonas ppk1* mutant is due to in the *E. coli* genome there is only one gene orthologous to the PPK1 protein [12]. In contrast, in *Pseudomonas aeruginosa* PAO1 the presence of another ortholog (PPK2) can compensate the lost of *ppk1* gene, in fact, despite the absence of detectable PPK1 activity (<1% of wild type), *Δppk1* mutants still possess as much as 20% of polyP of the wild type levels [12].

**Figure 2:**
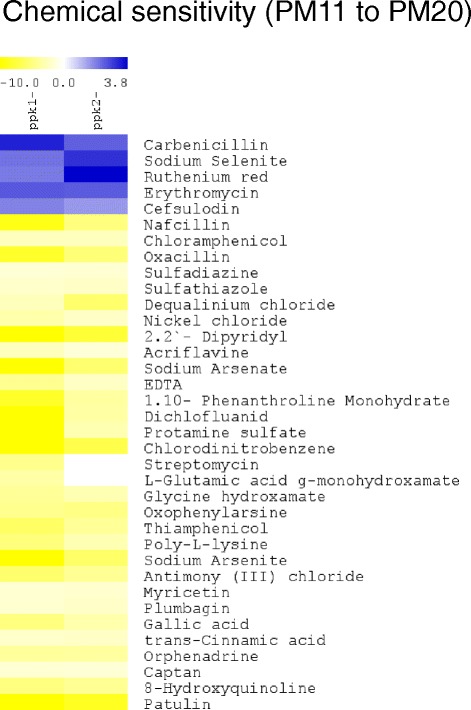
PolyP kinases (*ppk1* and *ppk2*) mutants from *P. aeruginosa* PAO1 are susceptible to multiple antibiotics. Clustering analysis of chemical sensitivity tests (PM11-20) from *P. aeruginosa* PAO1 *ppk1* and *ppk2* mutants. Gain (blue) and lost (yellow) phenotypes were standardized by dividing the respiration values of the mutants by the respective values of the wild type strain, for each phenotype.

Our results show that the majority of phenotypic changes observed were coincident in *ppk1* and *ppk2* mutants (Figure 1, Table 1). We found only few differences among phenotypes between both *ppk* mutants (Additional file 2: Tables S1-S4). We were able to identify novel phenotypes for polyP synthesis mutants in *P. aeruginosa* PAO1 and found that both mutants are susceptible to multiple antibiotics (Figure 2).

Interestingly, we observed susceptibility to various antibiotic families with different mechanisms of action such as Penicillins (Nafcillin, Oxacillin), Ansamycins (Streptomycin), Sulfonamides (Sulfadiazine, Sulfathiazole) and Others (Chloramphenicol, Thiamphenicol). To confirm this finding, we used two additional well-established methods for antimicrobial susceptibility testing. The results from the M.I.C.Evaluator™ (Oxoid) and Etest® (Biomeriux) corroborated the susceptibility of *ppk* mutants observed in the PM results (Additional file 2: Table S5). The minimal inhibitory concentration (MIC) parameters for both *ppk* mutants were particularly interesting for the antibiotics Rifampicin, Imipinen and Ciprofloxacin, either because *Pseudomonas* is intrinsically resistant or because recent strains highly resistant to these antibiotics have been found recently [20]. It should be noted that concentrations of antibiotics used in the PM experiments are set according to the minimal inhibitory concentration for *E. coli*. It is possible that other antibiotic susceptibilities have not been detected since both the control strain and the mutants were resistant.

Taking into account that various bacterial regulatory genes that participate in complex regulatory networks have been reported to influence both virulence and antibiotic resistance [20], polyP levels could affect regulators that control both antibiotic resistance and virulence.

Finally, our results support the recent finding that bacterial persistence, a phenomenon in which isogenic populations of antibiotic-sensitive bacteria produce rare cells that transiently become multidrug tolerant, is affected in *ppk1* deficient strain [21]. This small fraction of cells that grow slowly explains bacterial antibiotic tolerance because the cellular targets disturbed by lethal antibiotics are much less vulnerable in slow-growing than in fast-growing cells. This is important because the presence of persister cells has been suggested to be one of the main reasons for failures in the treatment of these chronic diseases. Indeed, clinical isolates of *Pseudomonas aeruginosa* from cystic fibrosis patients show an increase in high persistence mutants the longer that these isolates remain in the host. This indicates that persistence plays a major role in the failure to remove these bacterial populations from the cystic fibrosis lung [20].

Consequently, polyP synthesis, and particularly PPK1, in bacterial pathogens exhibits a potential target for antimicrobial drug design because combines the reduction of bacterial virulence and persistence, while simultaneously increasing susceptibility to antibiotics as we describe here by using PM technology. This could positively impact the host health for clearing the bacterial infection. Considering that no PPK1 homologs have been identified in higher-order eukaryotes [22], our phenotypic sensibility results highlight the importance of polyP synthesis as a putative target for the treatment of this opportunistic bacterium.

## Additional files

Additional file 1: Figure S1. Consensus graphical profile of metabolic and sensitivity tests of *P. aeruginosa ppk1* (A) and *ppk2* (B) mutants. Significant changes are enclosed in boxes. Yellow indicates that respiration rate of the wild type and mutant strains were similar. Red indicates faster respiration rate of the wild type (lost phenotype). Green indicates faster respiration rate of the mutant (gain phenotype). The quantitative difference values are shown in Table 1 and Supplementary tables. **Figure S2.** Clustering analysis of metabolic tests (PM1-PM8) and pH response (PM10) from *P. aeruginosa* PAO1 *ppk1* and *ppk*2 mutants. Gain (blue) and lost (yellow) phenotypes were standardized by dividing the respiration value of the mutants by the value of the wild type strain, for each phenotype. The results are shown separately for the different categories: carborn (C) sources, nitrogen (N) sources, phosphorus (P) and sulfur sources (S), peptide nitrogen (N) sources and pH response. **Figure S3.** Venn diagrams of phenotypic microarrays results from polyP synthesis mutants from *E. coli* K12 and *P. aeruginosa* PAO1. The numbers indicate the total phenotypes gained (A) or lost (B) between *P. aeruginosa ppk1* and *ppk2* mutants and *E. coli* Δ*ppk1* mutant. Phenotypic microarray results from *E. coli* Δ*ppk1* mutant were performed in a previous work (Unpublished results). Additional file 2: Table S1. Summary of *P. aeruginosa ppk1* mutant gain and lost phenotypes. **Table S2.** Summary of *P. aeruginosa ppk2* mutant gain and lost phenotypes. **Table S3.** Summary of unique *P. aeruginosa ppk1* mutant gain and lost phenotypes. **Table S4.** Summary of unique *P. aeruginosa ppk2* mutant gain and lost phenotypes. **Table S5.** Summary of antimicrobial susceptibility testing (M.I.C.Evaluator™(Oxoid) and Etest® (Biomeriux) for *P. aeruginosa* *ppk1* and *ppk2* mutant strains.

## Abbreviations

PPK: Polyphosphate kinase
PolyP: Polyphosphate
MDR: Multidrug resistance
PM: Phenotype Microarray (Biolog)
PCR: Polymerase chain reaction
MIC: Minimal inhibitory concentration
Pappk1: Polyphosphate kinase 1 from *Pseudomonas aeruginosa* PAO1
