# Supplementary material for: Multiple antibiotic susceptibility of polyphosphate kinase mutants (ppk1 and ppk2) from Pseudomonas aeruginosa PAO1 as revealed by global phenotypic analysis
Source: Biol Res. 2015 Apr 25;48(1):22. doi: 10.1186/s40659-015-0012-0 (PMC4424552; doi:10.1186/s40659-015-0012-0)
Supplement: Additional file 1: Figure S1. — Consensus graphical profile of metabolic and sensitivity tests of P. aeruginosa ppk1 (A) and ppk2 (B) mutants. Significant changes are enclosed in boxes. Yellow indicates that respiration rate of the wild type and mutant strains were similar. Red indicates faster respiration rate of the wild type (lost phenotype). Green indicates faster respiration rate of the mutant (gain phenotype). The quantitative difference values are shown in Table 1 and Supplementary tables. Figure S2. Clustering analysis of metabolic tests (PM1-PM8) and pH response (PM10) from P. aeruginosa PAO1 ppk1 and ppk2 mutants. Gain (blue) and lost (yellow) phenotypes were standardized by dividing the respiration value of the mutants by the value of the wild type strain, for each phenotype. The results are shown separately for the different categories: carborn (C) sources, nitrogen (N) sources, phosphorus (P) and sulfur sources (S), peptide nitrogen (N) sources and pH response. Figure S3. Venn diagrams of phenotypic microarrays results from polyP synthesis mutants from E. coli K12 and P. aeruginosa PAO1. The numbers indicate the total phenotypes gained (A) or lost (B) between P. aeruginosa ppk1 and ppk2 mutants and E. coli Δppk1 mutant. Phenotypic microarray results from E. coli Δppk1 mutant were performed in a previous work (Unpublished results). [file 40659_2015_12_MOESM1_ESM.pdf]

A

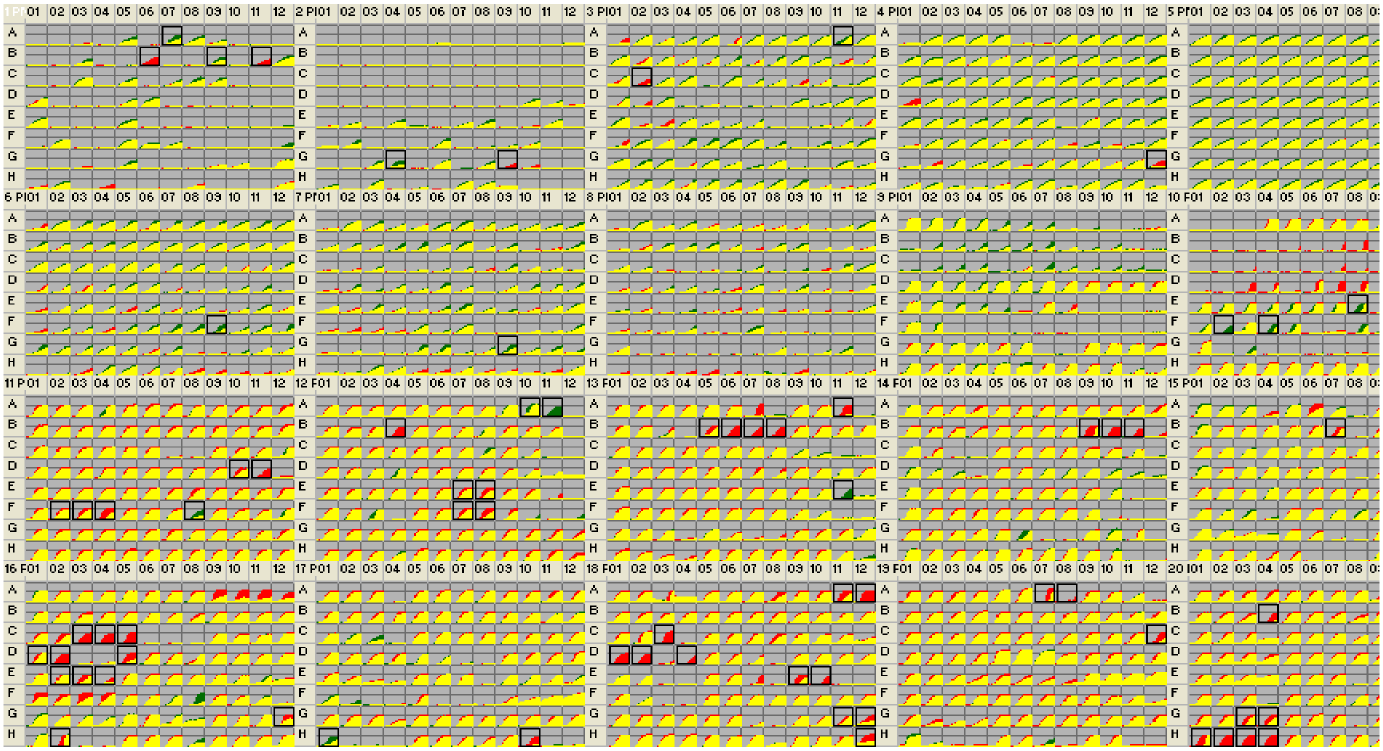

B

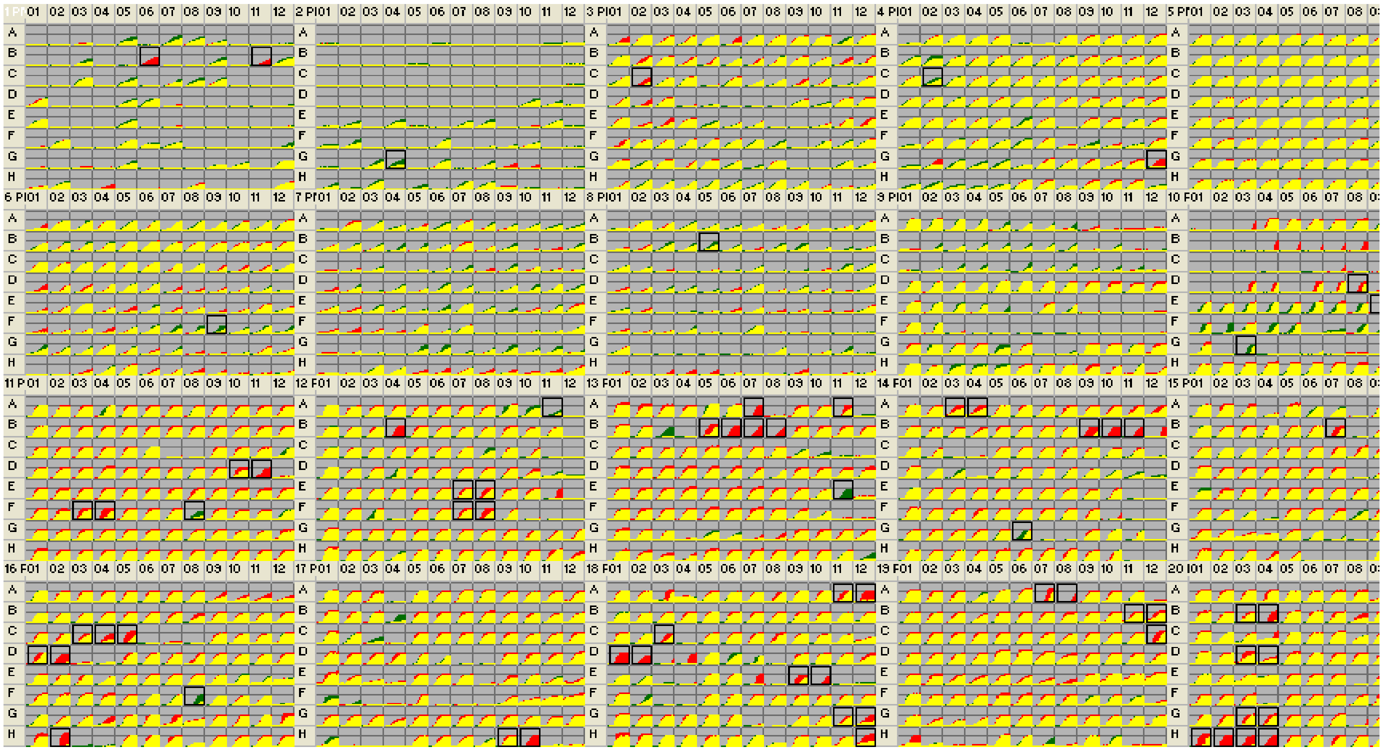

Figure S1

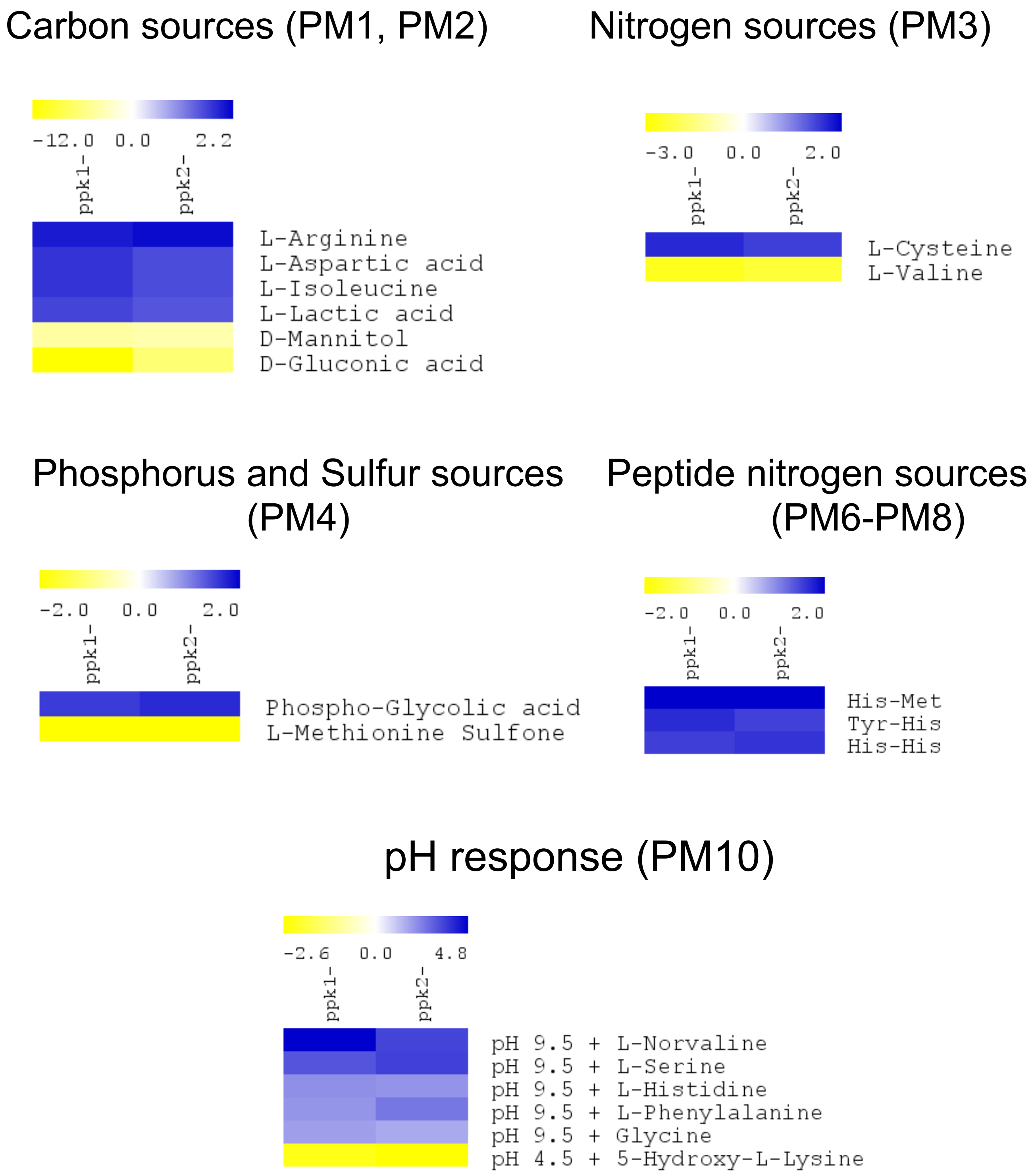

Carbon sources (PM1, PM2)
-12.0 0.0 2.2
ppk1-
ppk2-
L-Arginine
L-Aspartic acid
L-Isoleucine
L-Lactic acid
D-Mannitol
D-Gluconic acid
Nitrogen sources (PM3)
-3.0 0.0 2.0
ppk1-
ppk2-
L-Cysteine
L-Valine
Phosphorus and Sulfur sources (PM4)
-2.0 0.0 2.0
ppk1-
ppk2-
Phospho-Glycolic acid
L-Methionine Sulfone
Peptide nitrogen sources (PM6-PM8)
-2.0 0.0 2.0
ppk1-
ppk2-
His-Met
Tyr-His
His-His
pH response (PM10)
-2.6 0.0 4.8
ppk1-
ppk2-
pH 9.5 + L-Norvaline
pH 9.5 + L-Serine
pH 9.5 + L-Histidine
pH 9.5 + L-Phenylalanine
pH 9.5 + Glycine
pH 4.5 + 5-Hydroxy-L-Lysine

Figure S2

A

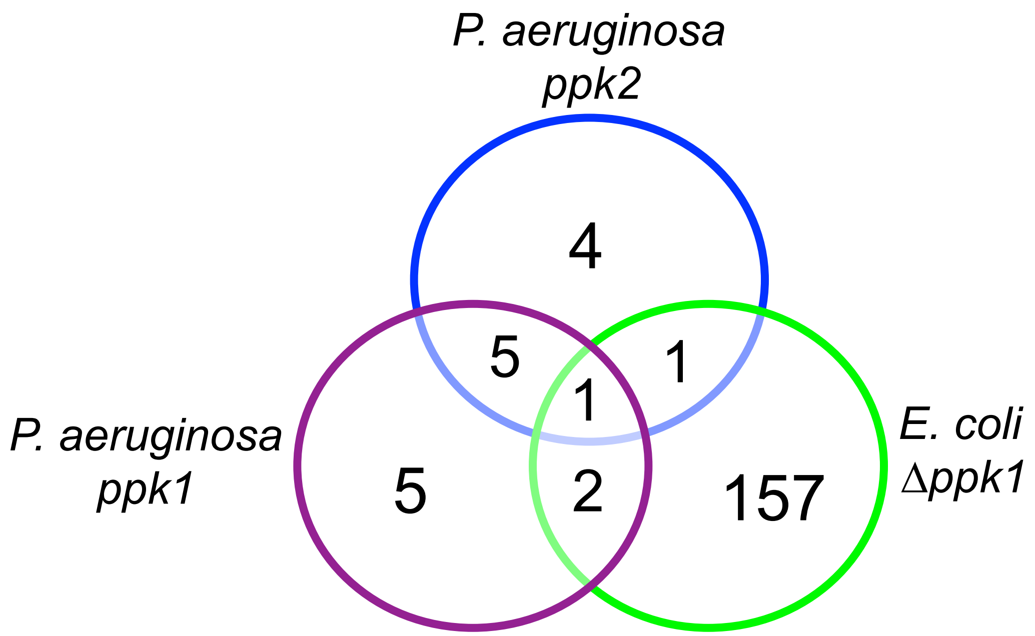

P. aeruginosa
ppk2
4
5
1
1
P. aeruginosa
ppk1
5
2
157
E. coli
Δppk1

B

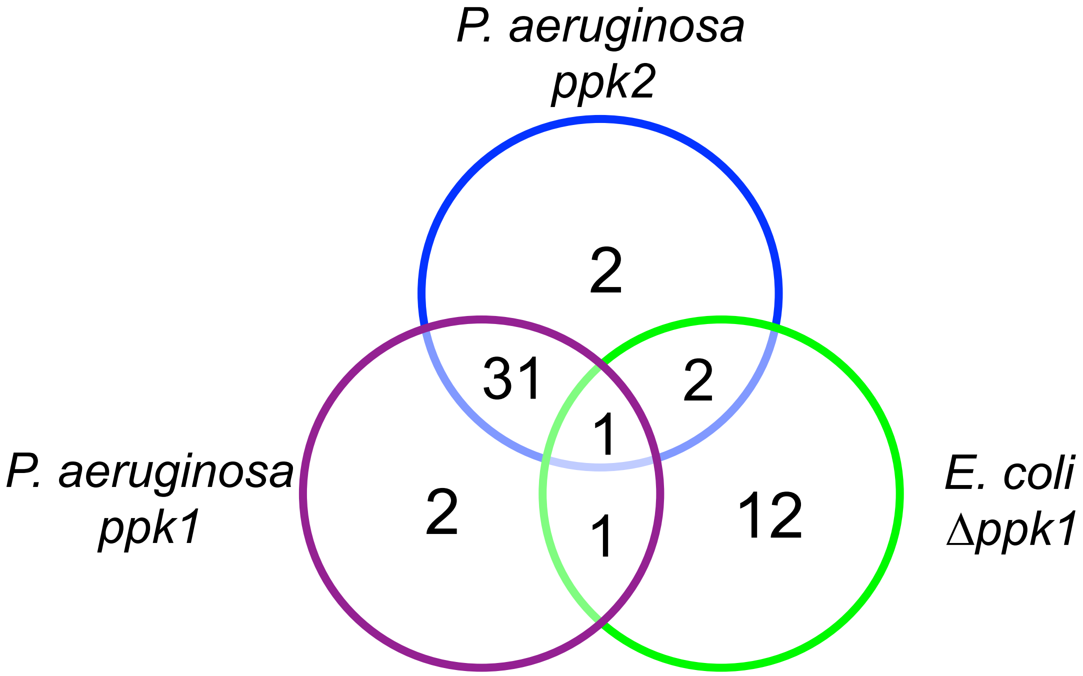

P. aeruginosa
ppk2
2
31
2
1
P. aeruginosa
ppk1
2
1
12
E. coli
Δppk1

Figure S3
