# Supplementary material for: Multiple antibiotic susceptibility of polyphosphate kinase mutants (ppk1 and ppk2) from Pseudomonas aeruginosa PAO1 as revealed by global phenotypic analysis
Source: Biol Res. 2015 Apr 25;48(1):22. doi: 10.1186/s40659-015-0012-0 (PMC4424552; doi:10.1186/s40659-015-0012-0)
Supplement: Additional file 2: Table S1. — Summary of P. aeruginosa ppk1 mutant gain and lost phenotypes. Table S2. Summary of P. aeruginosa ppk2 mutant gain and lost phenotypes. Table S3. Summary of unique P. aeruginosa ppk1 mutant gain and lost phenotypes. Table S4. Summary of unique P. aeruginosa ppk2 mutant gain and lost phenotypes. Table S5. Summary of antimicrobial susceptibility testing (M.I.C.Evaluator™(Oxoid) and Etest® (Biomeriux) for P. aeruginosa ppk1 and ppk2 mutant strains. [file 40659_2015_12_MOESM2_ESM.pdf]

Supplementary Tables and
Caption to Supplementary Figures

Table S1. Summary of *P. aeruginosa ppk1* mutant gain and lost phenotypes.

Test	Difference	Mode of action
	**Gain phenotype**	
L-Aspartic acid	68	C-Source, amino acid
L-Lactic acid	64	C-Source, amino acid
L-Arginine	50	C-Source, carboxylic acid
His-Met	61	N-Source, peptide
Tyr-His	53	N-Source, peptide
L-Cysteine	51	N-Source, amino acid
pH 9.5 + Glycine	69	pH, deaminase
pH 9.5 + L-Phenylalanine	66	pH, deaminase
pH 9.5 + L-Serine	61	pH, deaminase
Carbenicillin	158	Wall, lactam
Cefsulodin	80	Wall, cephalosporin
Ruthenium red	78	Respiration, mitochondrial Ca^{2+} porter
Erythromycin	67	Protein synthesis, 50S ribosomal subunit, macrolide
	**Lost phenotype**	
D-Gluconic acid	-112	C-Source, carboxylic acid
D-Mannitol	-68	C-Source, carbohydrate
L-Isoleucine	-57	C-Source, amino acid
L-Valine	-60	N-Source, amino acid
L-Methionine Sulfone	-69	S-Source, organic
Orphenadrine	-78	Anti-cholinergic
Patulin	-534	Microtubulin polymerization inhibitor, antifungal
2,2’-Dipyridyl	-401	Chelator, lipophilic
Sodium Arsenite	-332	Toxic anion
Sodium Arsenate	-305	Toxic anion, P04 analog
Dichlofluanid	-271	Fungicide, phenylsulphamide
Thiamphenicol	-269	Protein synthesis, amphenicol
Chloramphenicol	-209	Protein synthesis, amphenicol
Chlorodinitrobenzene	-182	Oxidizes sulfhydryls, depletes glutathione
8-Hydroxyquinoline	-181	Chelator, lipophilic
Nafcillin	-163	Wall, lactam
Antimony (III) chloride	-158	Toxic cation
1,10-Phenanthroline	-152	Chelator, lipophilic
monohydrate	-152	Fungicide, carbamate
Captan	-148	Folate antagonist, PABA analog
Sulfathiazole	-141	Respiration, ionophore, H^+
Gallic acid	-140	Protein synthesis, 30S ribosomal subunit,
Streptomycin	-140	aminoglycoside
Poly-L-lysine	-129	Membrane, detergent, cationic
Protamine sulfate	-127	Membrane, nonspecific binding
Sulfadiazine	-124	Folate antagonist, PABA analog
Oxacillin	-113	Wall, lactam
Myricetin	-113	DNA & RNA synthesis, polymerase inhibitor
Nickel chloride	-110	Toxic cation
Diamide	-106	Oxidizes sulfhydryls, depletes glutathione
Plumbagin	-99	Oxidizing agent
Oxophenylarsine	-98	Tyrosine phosphatase inhibitor
Trans-Cinnamic acid	-67	Respiration, ionophore, H^+
EDTA	-66	Chelator, hydrophilic
Glycine hydroxamate	-63	tRNA synthetase
L-Glutamic acid g-monohydroxamate	-63	tRNA synthetase

Table S2. Summary of *P. aeruginosa ppk2* mutant gain and lost phenotypes.

Test	Difference	Mode of action
	**Gain phenotype**	
L-Arginine	75	C-Source, amino acid
His-Met	56	N-Source, peptide
His-His	51	N-Source, peptide
Phospho-Glycolic acid	57	P-Source, organic
pH 9.5 + L-Histidine	84	pH, deaminase
pH 9.5 + L-Norvaline	62	pH, deaminase
Ruthenium red	110	Respiration, mitochondrial Ca^{2+} porter
Sodium Selenite	82	Toxic anion
Erythromycin	80	Protein synthesis, 50S ribosomal subunit, macrolide
Carbenicillin	71	Wall, lactam
Carbenicillin	40	Wall, lactam
	**Lost phenotype**	
D-Gluconic acid	-106	C-Source, carboxylic acid
D-Mannitol	-57	C-Source, carbohydrate
L-Valine	-58	N-Source, amino acid
L-Methionine Sulfone	-95	S-Source, organic
pH 4.5 + 5-Hydroxy-L-Lysine	-61	pH, decarboxylase
Patulin	-495	Microtubulin polymerization inhibitor, antifungal
2,2'-Dipyridyl	-397	Chelator, lipophilic
Sodium Arsenate	-321	Toxic anion, P04 analog
Sodium Arsenite	-242	Toxic anion
8-Hydroxyquinoline	-230	Chelator, lipophilic
Thiamphenicol	-193	Protein synthesis, amphenicol
Chlorodinitrobenzene	-187	Oxidizes sulfhydryls, depletes glutathione
Chloramphenicol	-173	Protein synthesis, amphenicol
Nafcillin	-172	Wall, lactam
1,10-Phenanthroline	-171	Chelator, lipophilic
monohydrate	-161	Tyrosine phosphatase inhibitor
Oxophenylarsine	-153	Toxic cation
Antimony (III) chloride	-146	Anti-cholinergic
Orphenadrine	-142	Folate antagonist, PABA analog
Sulfathiazole	-140	DNA intercalator, inhibits RNA
Acriflavine	-136	tRNA synthetase
Glycine hydroxamate	-130	Wall, lactam
Oxacillin	-122	DNA & RNA synthesis, polymerase inhibitor
Myricetin	-119	Fungicide, phenylsulphamide
Dichlofluanid	-118	Oxidizing agent
Plumbagin	-116	DNA intercalator, inhibits RNA
Proflavine	-115	Respiration, ionophore, H^+
Gallic acid	-113	Fungicide, carbamate
Captan	-109	Membrane, nonspecific binding
Protamine sulfate	-107	Folate antagonist, PABA analog
Sulfadiazine	-102	Ion channel inhibitor, K^+
Dequalinium chloride	-93	Respiration, ionophore, H^+
2,4-Dinitrophenol	-90	Respiration, ionophore, H^+
trans-Cinnamic acid	-88	Chelator, hydrophilic
EDTA	-80	Toxic cation
Nickel chloride	-63	Membrane, detergent, cationic
Poly-L-lysine		

Table S3. Summary of unique *P. aeruginosa ppk1* mutant gain and lost phenotypes.

Test	Difference	Mode of action
	**Gain phenotype**	
L-Aspartic acid	68	C-Source, amino acid
L-Lactic acid	64	C-Source, amino acid
Tyr-His	53	N-Source, peptide
L-Cysteine	51	N-Source, amino acid
pH 9.5 + Glycine	69	pH, deaminase
pH 9.5 + L-Phenylalanine	66	pH, deaminase
pH 9.5 + L-Serine	61	pH, deaminase
Cefsulodin	80	Wall, cephalosporin
	**Lost phenotype**	
L-Isoleucine	-57	C-Source, amino acid
Streptomycin	-140	Protein synthesis, 30S ribosomal subunit, aminoglycoside
Diamide	-110	Oxidizes sulfhydryls, depletes glutathione
L-Glutamic acid g-monohydroxamate	-63	tRNA synthetase

Table S4. Summary of unique *P. aeruginosa ppk2* mutant gain and lost phenotypes.

Test	Difference	Mode of action
	**Gain phenotype**	
His-His	51	N-Source, peptide
Phospho-Glycolic acid	57	P-Source, organic
pH 9.5 + L-Histidine	84	pH, deaminase
pH 9.5 + L-Norvaline	62	pH, deaminase
Sodium Selenite	82	Toxic anion
Carbenicillin	71	Wall, lactam
	**Lost phenotype**	
pH 4.5 + 5-Hydroxy-L-Lysine	-61	pH, decarboxylase
Acriflavine	-140	DNA intercalator, inhibits RNA
Proflavine	-116	DNA intercalator, inhibits RNA
Dequalinium chloride	-102	Ion channel inhibitor, K^+
2,4-Dinitrophenol	-93	Respiration, ionophore, H^+

Table S5. Summary of antimicrobial susceptibility testing (M.I.C.Evaluator™ (Oxoid) and Etest® (Biomeriux) for *P. aeruginosa* ppk1 and *ppk2* mutant strains.

	Antibiotics[1]									
**Strain**	**E**	**IPM**	**CIP**	**VA**	**CN**	**AMC**	**SM**	**CL**	**KM**	**RI**
**PAO1 WT**	16	2	0.75	>256	1	>256	8	>256	32	>32
**PAO1 *ppk1***	16	0.5	0.12	>256	1	>256	3	32	24	16
**PAO1 *ppk2***	16	0.5	0.12	>256	1	>256	3	32	32	3

[1] MIC (Minimal inhibitory concentration) parameter for antibiotics Erythromycin (E), Imipenen (IPM), Ciprofloxacin (CIP), Vancomycin (VA), Gentamicin (CN), Amoxicillin (AMC), Streptomycin (SM), Chloramphenicol (CL), Kanamycin (KM) and Rifampicin (RI).

Supplementary Figures

Figure S1. Consensus graphical profile of metabolic and sensitivity tests of *P. aeruginosa ppk1* (A) and *ppk2* (B) mutants.

Significant changes are enclosed in boxes. Yellow indicates that respiration rate of the wild type and mutant strains were similar. Red indicates faster respiration rate of the wild type (lost phenotype). Green indicates faster respiration rate of the mutant (gain phenotype). The quantitative difference values are shown in Table 1 and Supplementary tables.

Figure S2. Clustering analysis of metabolic tests (PM1-PM8) and pH response (PM10) from *P. aeruginosa* PAO1 *ppk1* and *ppk*2 mutants.

Gain (blue) and lost (yellow) phenotypes were standardized by dividing the respiration value of the mutants by the value of the wild type strain, for each phenotype. The results are shown separately for the different categories: carborn (C) sources, nitrogen (N) sources, phosphorus (P) and sulfur sources (S), peptide nitrogen (N) sources and pH response.

Figure S3. Venn diagrams of phenotypic microarrays results from polyP synthesis mutants from *E. coli* K12 and *P. aeruginosa* PAO1.

The numbers indicate the total phenotypes gained (A) or lost (B) between *P. aeruginosa ppk1* and *ppk2* mutants and *E. coli* Δ*ppk1* mutant. Phenotypic microarray results from *E. coli* Δ*ppk1* mutant were performed in a previous work (Unpublished results).
